# Supplementary material for: Are carbon emissions trading and green financial instruments synergistic? -Comprehensive quantitative research based on content analysis
Source: PLoS One. 2024 Mar 7;19(3):e0298601. doi: 10.1371/journal.pone.0298601 (PMC10919601; doi:10.1371/journal.pone.0298601)
Supplement: S1 Data — (ZIP) [file pone.0298601.s001.zip › Data/地级市Z_F工作报告.zip!/║╙─╧/σo╤⌠╩╨/└┤╫╘╣1⁄2╓┌║┼í╛┬φ┐╦╩2╛▌═°í┐_2006.docx]

各位代表：

现在，我代表市人民政府，向大会作政府工作报告，请予审议，并请各位政协委员提出意见。

一、2005年及“十五”工作回顾

2005年，市人民政府在省委、省政府和市委的正确领导下，坚持以科学发展观统领经济社会发展全局，积极构建和谐社会，深入实施以工兴市战略、科教兴濮战略、开放带动战略、可持续发展战略和品牌城市战略，在经济建设、政治建设、文化建设和社会建设等方面都取得了显著成绩，圆满完成了市五届人大二次会议确定的各项目标任务。

——国民经济平稳健康发展。初步统计，全市生产总值达到398亿元，比上年增长14．5％，比全省平均增幅高0．4个百分点。其中，第一产业增长7．5％，第二产业增长17．4％，第三产业增长11．5％。

——工业经济速度效益同步提高。全市规模以上工业增加值达到177．5亿元，比上年增长，19．5％。综合效益指数达到217％，提高57个百分点，创历史最高水平；实现销售收入500亿元，增长34．5％；实现利税71亿元，增长73．2％；盈亏相抵后实现利润46．3亿元，增长94．5％。

——农村经济全面发展。全市粮食总产达到202．4万吨，比上年增长3％。其中夏粮总产118．8万吨，增长3．6％；秋粮总产83．6万吨，增长1．9％。全市优质专用小麦、水稻、玉米分别占总播种面积的53．2％、66．4％和79．7％，经济作物面积达到213．7万亩；全年肉、蛋、奶产量分别增长13．4％、8．3％和47．9％。南乐县被评为“全国畜牧发展十佳示范县”。、农田水利基本建设取得了新进步，荣获全省“红旗渠精神杯”市级杯六连冠。加强林业生态建设，全市农田林网控制率和路沟渠绿化率达到95％和98％。

——重点项目建设实现突破。全市全社会固定资产投资完成145亿元，比上年增长23．3％。其中地方完成110亿元，增长44％。全市150个重点项目，有106个竣工投产，完成投资92亿元。一批关系濮阳发展大局的重点项目建设实现了新突破。林纸一体化项目年产10．8万吨杨木化机浆、中原大化年产3万吨三聚氰胺等一批项目建成投产。国电2x20万千瓦热电项目1#机组基本具备发电条件。大广高速公路濮阳段、中原大化50万吨甲醇、同力100万吨水泥粉磨站、范县陆集移民建镇等项目建设进展顺利。德商高速公路范县段工可报告通过专家评审。濮范高速公路完成勘测定界等工作。备受广大市民关注的城市用水黄河取水口改建工程开工建设。中原乙烯30万吨改造、2x35万千瓦燃气发电、盐化工等一批投资超10亿元的大项目正在积极做前期准备。年初向全市人民承诺的12件实事，完成10件，2件跨年度实事完成了计划进度。

——改革开放取得新成绩。国有企业改革稳步推进，全市177家国有企业改制面达到96％。完成了乡镇机构改革和国有粮食购销企业改革任务，其他各项改革正在按计划顺利推进。招商引资取得新成绩，全市共引进资金30．3亿元，增长29．7％。其中引进内资27．4亿元，增长33．4％，外资3681万美元，增长25．8％。外贸进出口总额达到1．7亿美元，增长32％。其中外贸出口达到1．6亿美元，增长57％。全年新签对外承包工程和外派劳务合同额3．3亿美元，完成营业额2．4亿美元，外派人数4130入，3项指标均居全省首位。市场繁荣活跃，全市社会消费品零售总额达到97．5亿元，比上年增长14．1％。

——非公有制经济发展势头强劲。全市非公有制经济实体达到9．2万家，从业人员55．2万人。规模以上非公有制工业企业达到432家，其中“双超”企业由上年的13家增加到19家。全市非公有制经济增加值达到180亿元，比上年增长28．5％；实现营业收入593．6亿元，增长19．6％；交纳税金7．3亿元，增长9．6％。非公有制经济占全市经济总量的45％。

——财政实力进一步增强。全市财政一般预算收入完成15．3亿元，比上年增长28．9％。一般预算支出完成34．8亿元，增长36．8％。金融机构各项存款余额达到307．6亿元，比年初净增26．5亿元。各项贷款余额达到143．9亿元，净增14亿元。与国家开发银行签订开发性金融合作协议，给予我市信用额度6亿元。

——城乡建设取得新成绩。完成了第三轮城市总体规划修编工作，为城市科学、有序发展提供了保障。西环路、北环路、京开大道北入市口拓宽改造、京开路濮水河桥等竣工通车，黄河路东延、五一路西延等跨年度道路建设进展顺利，光明街、重华街等10条背街小巷改造全部完成。建设了胜利路、黄河路、开州路污雨水泵站，完成了行政南小区污水泵站改造；10座垃圾中转站、11座公厕主体工程完工。地下水源给水工程基本建成，新增日供4万吨水源能力。濮水河截污工程开工建设。城市垃圾清运率和处理率均达到100％。城市绿化覆盖率达到41．4％，人均公共绿地面积14．6平方米。5个县城和小城镇建设取得了新成绩。文留镇被评为“全国文明镇”。全市城镇化率达到28．5％，比上年提高1．2个百分点。

——各项社会事业全面发展。全市组织实施各类科技项目145项，新增省级高新技术企业6家。清丰县被确定为“国家级科技富民强县专项行动试点县”。“普九”成果得到巩固，高等教育、高中阶段教育、职业教育和成人教育发展较快。累计改造中小学D级危房47．2万平方米，新建校舍37．3万平方米。其中2005年改造中小学D级危房26．5万平方米，新建校舍21万平方米，提前一年完成改造任务。广泛开展全民健身运动，成功承办了全国少儿乒乓球比赛。加强人口和计划生育工作，扎实开展“争先创优”活动，实现了低生育目标，全市人口自然增长率控制在5‰以下，低于全省平均水平。清丰县被评为“全国计划生育优质服务先进县”。以创建文化建设先进县为载体，全面加强农村文化建设，丰富了群众文化生活。首部《濮阳市志》正式出版，《濮阳年鉴》跨入全国先进行列。深入开展爱国卫生运动，巩固了国家卫生城市创建成果，被授予“全国无烟草广告城市”。完善公共卫生体系，华龙区被命名为“全国社区卫生服务示范区”，城乡居民的医疗卫生条件进一步改善。完成了有线电视网络整合。人民生活水平继续提高，全市城镇居民人均可支配收入达到8155元，比上年增长15％；农民人均纯收入达到2600元，增长18．6％。完成了第一次全国经济普查。安全生产工作进一步加强，荣获“全国安全生产宣传工作先进单位”称号。加大环境保护力度，完善资源有偿使用机制，合理保护和利用自然资源，资源节约型和环境友好型社会建设取得成效。切实加强审计执法监督，审计工作成效显著。土地管理、民族宗教、外事侨务、人防、地震、气象、价格管理、保险、邮电通信、残疾人事业、国防教育、民兵预备役等各项工作都取得了新的成绩。

——精神文明和民主法制建设进一步加强。大力推进精神文明创建活动，再次被命名为“全国创建文明城市工作先进城市”。深入开展“道德规范进万家、诚实守信万人行”活动，加强未成年人思想道德建设及大学生思想政治教育工作，城乡文明程度进一步提高。加强革命传统和理想教育，清丰单拐革命旧址被确定为“全国爱国主义教育示范基地”。继续推进基层民主政治建设，顺利完成第五届村委会换届选举。加强法制宣传教育，提高了全市人民的法律素质。自觉接受人大法律监督、工作监督和政协民主监督，认真办理人大代表议案、建议和政协提案。支持各民主党派、工商联民主监督、参政议政。认真贯彻《全面推进依法行政实施纲要》，全面落实行政执法责任制。廉政建设和反腐败斗争取得新成效。深入开展双拥工作，巩固军政军民团结。强化社会治安综合治理，公安大接访和命案攻坚活动成效显著，社会治安秩序保持稳定，被评为“全省社会治安综合治理先进市”。积极做好信访工作，制定完善了各类应急预案，建立健全应急机制，处理突发事件能力不断增强，社会大局保持稳定。

2005年全市经济社会发展各项目标的顺利实现，标志着我市“十五”计划主要任务的顺利完成。“十五”期间，全市累计完成生产总值1437亿元，是“九五”时期的1．7倍，年均增长11．7％；累计完成固定资产投资546亿元，是“九五”时期的1．96倍，年均增长15．5％；累计完成财政一般预算收入55．8亿元，是“九五”时期的1．7倍，年均增长19．3％。三次产业结构由“九五”末的21．5：54．5：24．0调整为“十五”末的17：59：24。2005年，在全国600多个城市综合评定中，我市入选中国特色魅力城市200强，中国社科院《2005年城市竞争力蓝皮书》对国内200个城市综合竞争力排名，我市列第77位居全省第3位。可以说，经过五年的努力，我市经济社会发展站在了一个新的战略起点上，进入了一个新的发展时期。

回顾“十五”尤其是2005年的工作，主要做法和体会是：

(一)坚持把结构调整作为发展经济的主线。结构不合理，自主发展能力不强，是我市经济发展中遇到的主要矛盾之一。对此，我们立足市情，坚持把结构调整作为促进科学发展的主导力量，通过发展推进调整，通过调整实现发展，不断促进产业结构优化升级。在工业方面，实行“抓大带小”两条腿走路。一方面，建立了地方政府与中央、省属企业联席会议工作机制，全力支持中央、省属大企业拉长链条，做大做强；另—方面，坚持“濮阳发展，产业支撑”的指导思想，用高新技术和先进适用技术改造提升传统产业，在把石化产业作为主导产业发展的同时，积极培育地方优势产业，支持三强集团、龙丰纸业等地方骨干企业膨胀规模，壮大实力。2005年全市新上投资超千万元的工业项目116个，其中超亿元的工业项目20个。全市“双超”企业发展到31家，其中有6家进入省百强企业行列，地方经济抗风险能力进一步增强。地方规模以上工业增加值占全市规模以上工业增加值的比重由上年的41．4％上升到44．6％。在农业方面，以调整结构促进农业增产、农民增收。大力发展特色高效农业，建成了尖椒、食用菌、冬枣、鲜切花、速生林等农林产品基地；培育壮大农业产业化龙头企业，全市各类产业化龙头企业达到376家，其中年销售额超亿元的10家，省级重点龙头企业4家。大力发展劳务经济，全市转移输出农村劳动力46．5万人，劳务收入达到23亿元。在服务业方面，加快现代服务业和新兴服务业发展，制定出台了《关于深化文化体制改革加快文化产业发展的意见》，全面落实加快旅游业发展的各项政策措施，文化产业和旅游产业得到发展。2005年，全市共接待游客217万人次，旅游收入2．7亿元。立足于建设三省交界处的现代物流商贸中心，加强物流基础设施和物流结点建设。加快厂房地产、金融、保险等服务业的发展步伐。在财政支出方面，加大了对“三农”、社会保障、科教文卫等社会事业的支持力度。2005年，全市用于改善农村生产生活条件支出5．4亿元，比上年增长77．8％；用于抚恤、社会福利救济和社会保障支出2．6亿元，增长34．5％；用于科教文卫等社会事业方面支出10．5亿元，增长39．9％，充分发挥了公共财政的职能作用。

(二)坚持把改革开放作为发展的动力。坚持用改革的办法解决发展中的矛盾和问题。针对制约经济社会发展的突出问题，迎难而上，克难攻坚，使改革不断向纵深发展。在国有企业改革方面，推出了一系列改革措施，因企制宜，一厂一策，全力推进。累计盘活有效资产1．1亿元，安置职工6014人，引进资金8805万元。按照省统一安排和部署，完成了乡镇机构改革任务。全市清退临时人员1313人，分流人员3289人，撤并清丰县王什乡、范县孟楼乡和濮阳县两门镇3个乡镇，全市精简乡镇领导干部438名，国有粮食购销企业改革全面完成，被授予“全省推进粮食企业改革卓有成效奖”。农村信用社体制改革进展顺利，六县(区)农村信用社全部获得专项央行票据，共获得票据额度7．5亿元。交通系统“三项改革”、建设系统“三项改革”、统计方法制度改革等顺利进行。坚持开放带动，把招商引资作为促进发展的主战略，创新招商方式，提高招商质量，取得了显著成效。在全省统一组织的豫港投洽会等招商活动中，我市共签约项目13个，总投资21亿元，其中合同项目5个，总投资6．4亿元，合同利用外资4．9亿元。在浙江萧山、台州和山东青岛进行的招商活动中，共签约项目64个，总投资47．8亿元。成功举办了世界张氏总会第二届恳亲大会，签约项目29个，总投资18．5亿元。

(三)坚持把壮大县域经济和非公有制经济作为经济发展的基石。认真落实和完善支持县域经济发展的各项政策措施，加大了对县域经济的扶持力度，引导各县(区)立足资源和比较优势，突出结构调整和优势产业，以招商引资为突破口，大力发展特色经济、民营经济、劳务经济，县域经济的活力和实力明显增强，全市各县区规模以上工业增加值增幅均在30%左右。围绕发展非公有制经济，落实政策，降低门槛，放宽市场准入，建立健全服务体系，加强对中小企业的融资担保、管理咨询、创业辅导等服务。进一步优化发展环境，大力开展招商引资，借助外力，推动非公有制经济加快发展。

(四)坚持把解决好人民群众最关心、最直接、最现实的利益问题作为工作的出发点。坚持以人为本，视群众利益高于一切。全面落实免征农业税、粮食直补、良种补贴、大型农机具补贴等支农惠农政策，全市农民减负9622万元，增收6095万元。高度重视农村饮水安全工程建设，投资1736万元，解决了10．1万人的安全饮水问题。努力解决农民就医难问题，建设了各县的传染病区；改造了11所乡镇卫生院，积极开展新型农村合作医疗试点工作，有24．3万人参加了新型农村合作医疗。大力做好救灾工作，按期完成了倒塌房屋、水毁工程的维修与重建，确保受灾群众“有饭吃、有房住、有衣穿、有病能及时治疗、学生正常上学”。切实加大扶贫工作力度，又有3．5万贫困人口解决和巩固了温饱。认真做好就业和社会保障工作。完善和落实了小额担保贷款、免费培训和税费减免等各项扶持政策，建立健全了就业服务体系和再就业援助制度，重点帮助城市困难群体再就业。全市城镇新增就业人员4．8万人，实现再就业1．97万人，其中“4050’’人员5900人。开工建设了市社会福利院，巩固了企业离退休人员基本养老金实行社会化发放和“低保”成果，城区居民的最低生活保障标准由143元提高到160元，并实现了应保尽保。

(五)坚持把优化环境作为促进发展的基础工程。坚持把环境作为经济发展的生命线来抓。在不断强化硬环境建设的同时，把更多的精力放在培育和增强软实力上，努力营造宽松灵活、公开透明的政策环境，规范、高效的服务环境，保障有力、依法有序的法制环境。转变政府职能，推行政务公开，简化工作程序，强化效能建设，为投资者提供“一站式”审批、“一条龙”服务。整顿和规范市场经济秩序，加大对“四乱”行为的查处力度。强化诚信意识，建立健全社会诚信体系，共铸诚信濮阳。加强社会治安综合治理，打造平安濮阳。

各位代表，“十五”及过去一年成绩的取得，是省委、省政府和市委正确领导的结果，是全市人民共同奋斗的结果。在此，我代表市人民政府向全市广大工人农民、知识分子、干部、人民解放军指战员、武警官兵、公安民警以及各界人士致以崇高的敬意，表示衷心的感谢!

在肯定成绩的同时，我们也要清醒地看到，当前我市经济发展中仍然存在一些突出矛盾和问题，主要是：经济结构单一，地方自主发展能力不强；农业基础薄弱，抗灾能力不强，农民增收的难度加大；城乡二元结构矛盾突出，城镇化进程滞后；体制机制还不完善，发展环境还有待于进一步优化等。此外，我们还面临原油减产、个别大企业因原材料供应不足而不能满负荷生产和市场变化等因素的不利影响。对于这些问题，我们要高度重视，采取积极有效措施，认真加以解决。

二、“十一五”发展的基本思路和奋斗目标

“十一五”期间是我市全面建设小康社会的关键时期。由于世界经济一体化的加快，科技进步日新月异，加速了国际产业结构调整和转移，沿海地区产业受资源、能源的约束，一些产业急剧向中西部转移。为了加快中部地区的发展，国家实施了中部地区崛起战略，在产业发展、重大基础设施建设等方面加大支持力度。这一大的形势给濮阳提供了不可多得的发展机遇。我们应该不失时机地抓住这个机遇期，乘势而上，加快发展。建市20多年来，我市经济社会健康稳定发展：各方面都有了新的积累，为加快发展提供了平台，特别是全市上下业已形成了团结奋进、干事创业的良好氛围，这些都有利于我市在新的战略起点上奋发有为，提速发展。因此，我们必须进一步增强责任感和紧迫感，珍惜和用好重要战略机遇期，在“十一五”期间新一轮经济社会发展中再上新台阶。

“十一五”时期，全市经济社会发展的基本思路是：坚持以邓小平理论和“三个代表”重要思想为指导，坚持以科学发展观统领经济社会发展全局，以经济建设为中心，以建设和谐社会为目标，大力实施以工兴市战略、科教兴濮战略、开放带动战略、人才强市战略、可持续发展战略和品牌城市战略，加速推进工业化、城镇化和农业现代化，转变经济增长方式，增强自主创新能力，推动经济转型，全面增强城市核心竞争力，把濮阳建设成为特色突出、优势明显，具有较强竞争力、辐射力和带动力的区域性中心城市。全市经济社会发展的主要奋斗目标是：生产总值年均增长13％以上；人均生产总值超过2万元；全社会固定资产投资年均增长15％以上，五年累计投资额突破1000亿元；社会消费品零售总额年均增长14％；出口总额累计达到9亿美元，实际利用外资累计达到2亿美元；“十一五”末，一、二、三产业比重达到13：59：28；非公有制经济增加值占生产总值的比重达到60％；全市文化产业增加值年均增长17％左右，占生产总值的比重达到4％左右；科技进步贡献率达到48％；单位生产总值能耗比“十五”末降低20％左右；财政一般预算收入略高于经济增长，力争五年实现翻一番；城镇居民人均可支配收入年均增长8％以上，农民人均纯收入年均增长6％以上；人口自然增长率控制在6．5‰以下；每年城镇新增就业3万人以上，城镇登记失业率控制在4．5％以内。

《濮阳市国民经济和社会发展第十一个五年规划纲要(草案)》及其说明已印发各位代表，请予审议。

三、2006年工作的主要目标和任务

2006年是“十一五”规划开局之年，开好头，起好步，关系濮阳的长远发展大局。今年我市经济社会发展的主要预期目标是：生产总值增长13％以上；地方财政一般预算收入增速略高于经济增长幅度；全部工业增加值增长15％；全社会固定资产投资增长18％；社会消费品零售总额增长14％；外贸出口总额增长20％，实际利用外资增长25％；居民消费价格上涨3％；城镇居民人均可支配收入增长9％，农民人均纯收入增长6％；单位生产总值能耗降低4％左右；人口自然增长率控制在6‰以下；城镇登记失业率制在4.5%以内，城镇新增就业人数4.1万人。

围绕上述目标，要重点抓好十个方面的工作：

(一)深入实施以工兴市战略，强力扶持八大优势产业发展壮大。近年来，我们在把石油和化工产业作为主导产业发展的同时，积极培育和扶持了一批市场潜力大、资源基础好、带动能力强的产业，初步形成了林纸林板、玻璃及电光源、食品加工、冶金建材、机械设备制造、纺织和电力等优势产业。2005年，八大产业增加值占全市全部工业增加值的96％，其中石油和化工产业占55.1％。可以说，八大产业在全市国民经济中举足轻重。根据濮阳经济发展的实际情况，为强力推进以上兴市战略，把濮阳经济做大做强，就必须发展支柱产业和优势产业，实现经济发展的产业化。为此，我们要把产业化发展作为工作重点，并确定将发展壮大八大产业作为调整经济结构、转变增长方式的重大战略举措和实施以工兴市战略、培育接续产业的基本内涵，采取措施强力推进。要明确“濮阳发展，产业支撑”的指导思想，以产业化发展作为我们工作的总抓手，强力扶持八大产业发展壮大，培育具有特色的产业集群、产业集聚区和产业带，发展一批自主创新能力强的大型企业集团，使我市的工业竞争力得到全面提升。在工作中，要按照“龙头带动、产业支撑、优化结构、集群发展”的思路，以培育产业龙头为突破口，发挥资源优势，拉长产业链条，扩大产业规模，形成产业基地，打造优势产业集群，促进产业优化升级，实现产业化发展，集中力量抓好128个投资超千万元的重点工业项目建设。要围绕八大产业，着力抓好以下项目建设：石油和化工产业：做好中原乙烯扩能改造前期工作，力争年内开工建设；加快中原大化煤化工项目建设，完成年产50万吨甲醇项目计划投资进度；市甲醇厂20万吨甲醇技改项目年内要开工建设，颖泰化工年产1万吨农药中间体项目和清丰凯利化工乙醇项目年内竣工投产；积极做好盐化工项目的前期工作。林纸林板产业：在抓好濮阳龙丰纸业制浆稳产的基础上，开工建设龙丰纸业20万吨造纸项目。玻璃及电光源产业：做好濮阳家雄灯饰二期工程续建；开工建设濮阳日佳灯业、兴富灯饰、中泰灯饰、中基灯业和台一照明等电光源项目。食品加工产业：搞好“中粮面业濮阳工业园”项目建设；抓好清丰先锋实业年产3000吨尖椒色素项目和源丰实业年产5000吨蒜粉加工项目；南乐福鑫肉业肉牛加工项目要调试稳产达产；河南伊利吉庆冷冻食品项目年内要建成投产。冶金建材产业：濮阳同力水泥年产100万吨水泥粉磨站争取一季度建成投产；做好林钢1#、2#高炉改造和年产3万吨精密铸件项目前期准备工作；做好濮耐2万吨非氧化物项目和南乐津华建材石膏砌块项目续建工作。机械设备制造产业：抓好中原总机厂抽油机(1000台／年)产能扩大和钻井泵(100台／年)规模制造，加快海华机械厂高压阀门、信宇工业园抽油杆项目扩能建设。纺织产业：抓好清丰永佳700台织机及羊毛衫生产线、濮阳庆南纺织三期改造项目建设。电力产业：国电2x20万千瓦热电项目2#机组年内建成投产；继续做好2x35万千瓦天然气发电工程前期工作；开工建设濮阳县秸秆发电项目，做好清丰县、南乐县秸秆发电项目前期准备。上述项目是我市产业化的重大项目，事关我市经济发展大局，市委、市政府将研究制定有关扶持政策、保障措施和责任制工作机制，确保产业化龙头项目建成。争取用2至3年的时间，使八大产业膨胀总量，成为濮阳的支柱产业。各级政府和部门务必高度重视，狠抓落实，推动项目建设顺利开展。市发改委、企业服务局、国资委、统计局等有关部门要加强监测、分析和指导，确保工业经济健康发展。

(二)建设社会主义新农村，全面推进“三农”工作。认真贯彻落实今年中央—号文件精神，坚持把“三农”工作作为重中之重，按照十六届五中全会提出的“生产发展、生活宽裕、乡风文明、村容整洁、管理民主”的20字方针要求，科学规划，扎实推进社会主义新农村建设。一要全面落实粮食直补等各项支农惠农政策，按照“多予少取放活”的要求，建立以工促农、以城带乡的长效机制，努力实现农业增效、农民增收。二要大力推进农业结构调整。稳步提高优质小麦、优质水稻、优质玉米的种植比重，提高粮食综合生产能力。加快农产品由初级加工向精深加工转变，拓展粮食增值空间。大力发展经济作物，进一步加强尖椒、花卉、无公害蔬菜等高效经济作物基地建设。大力发展畜牧养殖业，重点建设沿黄三县优质肉牛生产基地、“架子牛”生产基地和绿色奶业示范带。新建速生丰产林基地10万亩，推进林业产业化和林业生态建设。加快农业产业化发展步伐，重点扶持50家市级重点龙头企业，力争再有3至5家进入省级龙头企业行列，增强龙头企业的辐射带动能力。大力发展劳务经济，年内完成50万人劳务输出，增加农民非农业收入。三要加强农村基础设施建设。加大农村交通设施、水利基础设施及配套设施建设力度，加强黄河堤防和滩区基础设施建设，提高抗灾能力。鼓励农民投工投劳建设自己受益的小型农田水利，提高兴修小型农田水利设施补助标准，逐步建立农田水利建设用工新机制。四要大力发展农村社会事业。认真落实农村义务教育“两免一补”政策，巩固提高农村义务教育水平。加快农村文化、卫生、体育等公共基础设施建设，繁荣农村文化，丰富群众文化生活。五要加大扶贫工作力度，实施整村推进扶贫计划，年内解决和巩固温饱2．9万人。六要全面推进农村综合改革。巩固乡镇机构改革成果，确保乡镇机关事业人员只减不增，推进乡镇机构职能转变和服务水平提高。推进农村义务教育和县乡财政体制改革等。七要认真组织实施社会主义新农村建设示范行动。要在学习李连成，建设西辛庄式小康村的基础上，因地制宜，探索多种路子，实现建设社会主义新农村的良好开局。

（三）强力推进重点项目建设，千方百计扩大固定资产投资。

要把基础设施建设和基础产业发展放在重要位置，千方百计大上项目、上大项目、增强经济发展后劲。今年要着力抓好产业和基础设施方面的170个重点项目，概算总投资196亿元，年内计划完成投资97亿元。开工建设德商高速公路范县段、濮范高速公路、豫冀省界高速公路等大项目；年内大广高速公路濮阳段要建成通车；今年确定的农村新建和改造道路建设项目要全面完工。各级各有关部门要切实增强责任意识，认真落实重点项目责任制，从年初开始就要一个项目一个项目地抓落实，切实解决好项目核准、资金落实、征地拆迁、建设环境等问题，尽快掀起固定资产投资高潮。市重点项目稽察办公室要加大稽察监管力度，扎实推进项目建设进度。同时各级各部门要排出自己的重点项目，落实责任制，强力推进，增加新的经挤增长点。要按照国家、省的产业政策和投资方向，再调整充实一批市场潜力大、科技含量高、带动能力强的项目，积极对外招商，增强经济发展的持续动力。

今年市政府要办好与全市人民群众生产生活息息相关的12件实事。 （1）落实对农民的补贴政策。筹措6100万元资金，对种粮农民实施直接补贴、良种补贴和购买大型农机具补贴。落实好村经费和村干部报酬。 (2)增强农村义务教育保障能力。筹措5000万元资金，对农村义务教育阶段贫困家庭学生继续实施“两免一补”；按照建立长效机制要求，对新增农村中小学D级危房进行改造，实施农村中小学远程教育。 (3)改善人民群众医疗卫生条件。筹措1300万元资金，完成20个乡镇卫生院改造。筹措3700万元资金，抓好新型农村合作医疗改革，扩大试点覆盖面。各级财政对参合农民每人每年补贴标准由20元提高到40元。 (4)改善农民出行条件。筹措3000万元资金，改造和建设农村公路200公里。完善农村客运网络，新建10个乡级客运站。 (5)大力推进农村安全饮水工程建设。筹措2160万元资金，解决6万农民安全饮水问题。 (6)加快城市用水工程建设。筹措5000万元资金，完成渠村引黄闸改建工程，让广大市民吃上放心水，并解决三县两区农田灌溉用水问题。 (7)加快农村沼气建设。筹措3000万元资金，扶持农村沼气工程建设，新增沼气用户3万户。 (8)加快金堤河下游支沟口治理工程建设，筹措2000万元资金，再建设3座支沟口闸站，解决范县、台前县内涝问题。 (9)做好城镇就业再就业工作。筹措4800万元资金，扶持再就业工程，年内新增城镇就业4.1万人。 (10)加快农村富余劳动力转移。筹措240万元资金，充分利用市、县(区)两级各类培训资源，培训农村劳动力10万人，新转移农村富余劳动力6万人。 (11)加强城区教育基础设施建设。筹措3000万元资金，改造市子路小学等一批中小学校的基础设施，改建乙烯小学为市第十中学，开工建设市第九中学。 (12)完善城市基础设施。完成10条背街小巷改造，建设6座公厕、3座垃圾中转站，完成濮水河截污工程。

(四)加快改革开放步伐，为经济发展注入新的活力。要按照省政府要求，全面推进各项改革。一要深化国有企业改革。以产权制度改革为重点，加快国有企业改革步伐。完成市属16户重点国有工业企业的改革改制，全市国有工业企业改革基本完成，全面推开国有非工业企业改革。要建立国有资产有效监管体制，依法向企业派出监事会，完善现代企业制度，加强对国有资产的监管，确保国有资产保值增值。二要大力推进文化体制改革，加快发展文化产业。按照政企分开、政事分开和管办分离的原则，加快国有文化企业的公司制改造和经营性文化事业单位转企改制，建立现代企业运行机制。引导和鼓励社会资本兴办文化企业，鼓励发展民营文艺表演团体，尽快把文化产业打造成我市支柱产业。三要积极推进现代市场体系建设。进一步完善市场体系规划。继续扩大债券、信托融资规模。大力整顿和规范市场秩序，加快建设社会信用体系，树立濮阳信用形象。四要全面推进其他领域的改革。继续深化财税体制改革和农村信用社改革。完成建设系统“三项改革”。推进资源性产品价格改革。加快交通、水利、人事、卫生等领域的改革。要建立文化产业、服务业、物流业、能源消耗；县域经济监测、劳动力调查和外商投资统计等新的统计制度，做好规模以上工业基本单位名录库制度改革工作。

要坚持把招商引资作为加快濮阳发展的主战略来抓，进一步完善措施，强化激励机制，扩大引资规模。一要创新招商方式，提高招商实效。要大力推行委托招商、中介机构招商、网上招商等市场化招商做法，搞好专项和专业性招商。要认真组织参加国家和省举办的各类“商洽会”和“投洽会”等大型商贸活动，切实办好世界舜裔宗亲联谊会，组织好赴“环渤海经济圈”招商活动。把招大商、引大资作为一项硬任务，调整思路，克服困难，争取更多的世界500强企业落户濮阳。二要加强已签约重大项目的跟踪落实。继续抓好在“长三角”、“珠三角”、“世张”大会、浙江、山东等招商活动中所有签约项目的跟踪问效及后续管理工作，提高合同履约率。三要加强外经外贸工作。优化出口商品结构，多上高科技产品、终端产品和特色产品，增强出口竞争力。大力开拓外经工作，积极发展对外工程承包和劳务合作。

要毫不动摇地把发展非公有制经济作为强市富民的战略措施来抓，采取切实有效措施，认真落实国务院《关于鼓励支持和引导个体私营等非公有制经济发展的若干意见》，落实优惠政策，创造良好环境，提供优质服务，支持鼓励民营资本进入国家法律法规没有禁止进入的行业和领域，进入高新技术产业、基础性产业、现代服务业和公用事业领域，支持非公有制企业以各种方式参与国有企业的改造和重组。进一步完善激励措施，大力宣传和表彰先进典型，促进非公有制经济健康快速发展。

(五)加快城镇化步伐，推动城市经济和县域经济发展，一要认真编制和执行“十一五”城镇化发展规划。要以科学发展观为统领，高起点、高标准、高质量地编制出具有超前性、阶段性和科学性的城镇化发展规划。规划一经确定，必须严格按照规划做好城镇化各项工作。二要强化产、比支撑，增强城镇化发展的基础和动力。要加快城镇二、三产业发展，打造一批竞争力较强的产业集群、产业集聚区和产业带，使产业成为推进城镇化的动力源泉和物质基础。城市经济和县城经济是产业发展的重要载体，也是统筹城乡协调发展的重要平台，必须坚持城市经济和县域经济两手抓，促进城镇化发展；一方面，要大力发展城市经济。市发改委、国资委、商务局、企业服务局等部门要继续按照市委、市政府的安排部署，把发展城市经济摆上重要议事日程，切实担负起城市经济发展主力军的重要责任，成为市本级招商引资和增加新的经济增长点的主体。市高新区要进一步搞好工业园区建设，打造城市经济发展平台，积极引导市级产业项目进区发展。另一方面，要大力发展县域经济。进一步落实和完善支持县域经济发展的各项政策，加大扶持力度。各县要立足县情，准确定位，发挥优势，进一步完善县城基础设施和公共服务功能，打造特色产业园区，大力发展特色经济、非公有制经济和劳务经济，促进县域经济加快发展。三要降低门槛，增加城镇人口。要加快户籍制度改革步伐，在全市逐步推行按实际居住地进行登记的户籍管理制度。要继续清理限制农民进城的歧视性政策规定；对进城农民在就业、经商、子女入学等方面与城镇居民同等对待，促使人口由农村向城镇集中，劳动力从第一产业向二、三产业转移。四要扩大城市规模，完善服务设施。加强城镇基础设施和公共服务设施建设，重点搞好城镇水、电、气等各种管网的综合布局，加快园林绿化、污水处理等生态环境保护工程建设，完善教育、文化、广电、科技、医疗卫生、体育、旅游等公共服务设施。五要坚持区域性中心城市、县城、小城镇建设“三头并进”。继续大力实施品牌城市战略，巩固发展“六城二奖”创建成果，拉大城市框架，完善城市功能，提高城市品位和档次，进一步增强濮阳的影响力和竞争力。今年要完成黄河路东廷、建设路东延、开州路北延等城市道路拓宽延伸改造，对濮鹤高速引线至京开大道进行高标准改造，完成振兴路北延等3个城市建设项目的续建任务，提高城市道路、公共绿地绿化美化水平。要加大城市综合整治力度，治理脏乱差，依法清理乱搭乱建，维护城市市容环境。5个县城和小城镇建设要根居规划，完善功能，加快发展，要按照省政府要求，完成污水处理厂、生活垃圾处理场的建设进度。

(六)加强财税金融工作，更好地支持经济社会发展。要树立发展经济、培植财源的思想，大力培植税源，为经济建设提供保障 、依法强化税收征管，积极推行综合治税，堵塞“跑、冒、滴、漏”，做到应收尽收。严格执行“收支两条线”规定，进—步加强非税收入管理，重点要加强土地、广告、污水处理费、市政设施经营权转让等非税收入的收缴管里，积极争取中央、省财政转移支付，增加可用财力。要合理安排财政支出，保证农业、教育、科技、社会保障等法定和重点支出需要。要继续推进部门预算、国库集中收付、政府采购和政府投资评审等改革，严格支出管理。严禁大手大脚、铺张浪费，提高财政资金的使用效益。要认真贯彻国务院《财政违法行为处罚处分条例》，加大审计执法力度，提高审计质量。金融机构要突出信贷重点，用足用活政策，加强内部管理，增加信贷投入，确保重点企业、重点建设项目的资金需求。各级政府要加大对金融部门的支持力度，搞好服务，为金融机构创造良好的经营环境。

(七)加快经济转型，建设资源节约型和环境友好型社会。一要认真落实国务院《促进产业结构调整暂行规定》，把增强自主创新能力作为调整产业结构、转变增长方式、提高核心竞争力的中心环节来抓，坚持自主创新和技术引进相结合，加快创新体系建设，促进科技与经济的紧密结合，促进接续产业的发展，全面提升科技整体实力和产业技术水平。支持重点企业发挥主体作用，加快技术中心建设，加强关键领域的技术攻关，启动实施一批科技项目，争取形成一批自主知识产权。要进一步发挥消费对经济增长的拉动作用，大力发展服务业，扩大消费需求。抓好“万村千乡”市场工程的实施，加快农村消费市场建设。规范发展房地产业。围绕建设三省交界处商贸流通中心，积极引进国际、国内知名的大型商业企业集团投资或设立分支机构。要全面落实全市旅游产业发展大会精神，加大促销力度，加强行业管理，整合资源、打造旅游精品，提高旅游业发展水平，把旅游业培育成我市新的经济增长点。二要大力推进资源节约。要从严控制高耗能、高耗材、高耗水产业的发展，淘汰落后生产能力，依法关闭破坏资源和污染环境的企业。严格执行重点产品能耗限额标准，加强高耗能行业节能管理。全面清理整治砖瓦窑厂，逐步禁止生产实心粘土砖，积极推广新型墙体材料。加强国土资源管理，盘活存量建设用地提高土地集约利用水平。三要大力发展循环经济。编制并实施全市循环经济发展规划。加强生态工业园区和秸秆综合利用农业小区建设，推行清洁生产，发展余热利用，建设环境友好型企业。四要搞好环境保护和生态建设。按照《国家生态示范巨建设规划》要求，扎实推进生态示范区建设各项工作。加大重点污染流域、区域和重点污染企业综合治理。继续实施退耕运林工程，积极推进西部沙区、黄河滩区生态林建设。

(八)统筹兼顾，发展各项社，会事业。大力实施科教兴濮战略，整合优化科技资源配置，组织实施各类科技计划项目120项。申报国家科技型中小企业技术创新基金项目和省高新技术成果转化项目2项，新增省高新技术企业3家、民营科技企业35家。实施“科技富民工程”，完善农村科技服务体系以农村义务教育为重点，加大教育投入，提高农村中小学公用经费标准。加强城市中小学、普通高中建设，加快发展高等教育、职业教育，促进教育资源的均衡配置。加大对城乡文化设施的投入，抓好现有乡级文化站的巩固提高，新建13个达标文化站。加强文物保护工作。坚持不懈地开展“扫黄打非斗争”。加强体育基础设施建设，发展体育事业，在省十运会上争创住绩。积极推进城镇医疗卫生体制改革，继续开展医院管理年活动，抓好台前县、濮阳县新型农村合作医疗试点工作，大力发展农村医疗卫生事业和城市社区卫生服务，改善城乡居民的医疗卫生条件。以迎接国家卫生城市复检为重点，大力开展爱国卫生运动。做好人口和计划生育工作，深入开展优质服务活动，落实奖励扶助政策，继续治理性别比升高问题，稳定低生育水平，提高出生人口质量。按照中央和省要求，做好第二次全国农业普查工作。

(九)坚持以人为本，切实解决关系人民群众切身利益的问题。一要扩大就业。把扩大就业摆在突出位置，多渠道开发就业岗位，支持社区就业和灵活就业。继续做好高校毕业生、城镇新增劳动力和农村富余劳动力的就业工作。进一步完善落实就业和再就业的各项优惠政策，把小额贷款、税费减免等再就业优惠政策落到实处。按照省统一部署，国有企业下岗职工再就业扶持政策再延长三年。二要完善社会保障体系。加快建立就业与失业保险、城市居民最低生活保障工作的联动机制。完善城镇职工基本养老保险制度，确保企业离退休人员基本养老金按时足额发放。完善城市居民最低生活保障制度，实现动态管理下的应保尽保。认真解决进城务工人员和失地农民的社会保障问题，落实农村五保供养政策，切实解决农村特殊困难家庭的实际困难。三要稳步提高居民收入水平和生活质量。提高城镇中低收入者的收入水平，严格执行最低工资制度。按照国家和省政府的规定，改革公务员工资制度，规范津贴、补贴。认真清理建设领域拖欠工程款和农民工工资，全面实现清欠目标。四要进一步整顿和规范市场经济秩序，严厉打击制售假冒伪劣产品、商业欺诈等违法犯罪行为。五要加强安全生产和社会稳定工作。严格落实安全生产责任制，坚决遏制重特大事故的发生。认真做好信访工作，积极预防和妥善处置群体性事件。加强社会治安综合治理，完善社会治安防控体系，严厉打击各类刑事犯罪和经济犯罪，严防敌对势力和邪教组织渗透，建设平安濮阳，营造城乡居民安居乐业的社会环境。

(十)加强精神文明和民主法制建设，构建和谐社会。切实加强理想信念教育和思想道德建设，推进公民道德建设。加强和改进青少年思想道德建设以及大学生思想政治教育工作。深入开展群众性精神文明创建活动，扎实开展农村精神文明创建，积极开展文明城市、文明社区创建工作。坚持依法治市。深入开展普法教育，提高全民法律素质。自觉接受人大及其常委会的法律监督、工作监督和人民政协的民主监督，严格执行人大及其常委会的决议、决定，支持人大代表依法行使职权，认真办理人大代表议案、建议和政协提案。广泛听取各民主党派、工商联的意见和建议，充分发挥工会、共青团、妇联等人民团体的桥梁纽带作用。全面贯彻党的民族宗教政策，做好民族宗教工作。加强法律援助工作，依法保障妇女、未成年人和残疾人的合法权益。积极发展慈善事业。继续做好广播电视、外事侨务、地震、气象、价格管理、保险、邮电通信、残疾人事业工作。

积极支持部队和国防建设。深入开展国防教育，健全国防动员体系。关心和支持驻濮解放军和武警部队建设。抓好人民防空和民兵预备役工作。认真落实军人优待抚恤和退伍安置政策，做好军队转业干部、退役军人安置工作。深入开展争创“全国双拥模范城”活动，增强军政军民团结。

四、加强政府自身改革和建设

各级政府和政府各部门要进一步强化创新意识、服务意识、责任意识，切实加强政府自身改革和建设，努力做到为人民服务、对人民负责、受人民监督、让人民满意。

改革创新，加快政府职能转变。以转变政府职能为重点，大力推进政企分开、政资分开、政事分开、政府与市场中介组织分开。继续深化行政审批制度改革，进一步减少和规范行政审批事项。搞好经济调节和市场监管，更加注重社会管理和公共服务。依法管理和规范社会组织、社会事务，协调各种利益关系，促进社会公平和正义。完善突发性公共事件应急预案，提高预防和处置突发性公共事件的能力。完善政府责任目标考评制度，增加衡量科学发展、和谐社会建设等方面的指标，实施GDP能耗指标公报制度。增强改革的主动性，以改革促发展，向改革要效益。

执政为民，建设服务型政府。把实现好、维护好、发展好人民群众的根本利益作为政府工作的出发点和立足点，强化宗旨意识，提高服务质量，更好地为基层、企业和社会公众服务。积极整合行政资源，有效降低行政成本，切实提高行政效率。大力推进政务公开，在公共服务部门和单位全面推行办事公开制度，充分实现人民群众的知情权、参与权和监督权。推进电子政务建设，完善政府网站，及时公布政务信息。完善行政服务大厅功能，精简办事程序，切实履行服务承诺，真正做到高效便民，取信于民。

依法行政，建设法治政府。认真贯彻行政许可法和依法行政实施纲要，完善行政程序，规范行政行为，健全对行政权力的监督机制。依法规范行政执法主体，实行行政执法主体资格公告制度，全面推进行政执法责任制。进一步落实规范性文件管理规定和重大具体行政行为备案审查制度，认真做好行政复议工作，强化政府法制层级监督。完善行政管理决策机制，健全对涉及全局的重大事项决策的协商和协调机制，推进行政管理决策科学化、民主化。全面推行领导干部经济责任审计制度。加强行政效能监察，落实廉政建设责任制，深入开展反腐败斗争，建设廉洁政府。

求真务实，加强政风建设。坚持求真务实，多做打基础、管长远的工作，防止急功近利，克服官僚主义和形式主义。大力精简会议、文件，改进会锋、文风，严格控制和规范各种检查、评比、达标活动。厉行节约，反对浪费，建设节约型机关。大兴调查研究之风，掌握真实情况，解决实际问题。强化工作责任，加强督促检查，不折不扣地抓好落实。认真实施公务员法，加强公务员教育、管理和监督，建设高素质的公务员队伍。大力推行效能问责制，实施“治庸计划”，着力解决行政不作为、乱作为、不会作为问题，提高执政能力，努力加快濮阳发展步伐。

各位代表，我们面临的任务光荣而艰巨。让我们在省委、省政府和市委的领导下，高举邓小平理论和“三个代表”重要思想伟大旗帜，团结拼搏，锐意进取，扎实做好今年的工作，为开创我市“十一五”时期经济社会发展新局面而努力奋斗!
